# A rapid, accurate, and low-cost method for detecting *Mycobacterium tuberculosis* and its drug-resistant genes in pulmonary tuberculosis: Applications of MassARRAY DNA mass spectrometry

**DOI:** 10.3389/fmicb.2023.1093745

**Published:** 2023-02-23

**Authors:** Han Yang, Aifang Li, Liyun Dang, Tao Kang, Fei Ren, Jinbao Ma, Yong Zhou, Yuanli Yang, Jing Lei, Tao Zhang

**Affiliations:** ^1^College of Pharmacy, Xi’an Jiaotong University, Xi’an, China; ^2^Medical Transformation Centre, Xi'an Chest Hospital, Xi 'an Jiaotong University Health Science Center, Xi’an, China; ^3^Department of Laboratory Medicine, Xi'an Chest Hospital, Xi 'an Jiaotong University Health Science Center, Xi’an, China; ^4^Department of Tuberculosis, Xi'an Chest Hospital, Xi 'an Jiaotong University Health Science Center, Xi’an, China; ^5^Department of Reagent, Zhejiang Digena Diagnosis Technology CO., LTD, Zhejiang, China; ^6^Endoscopy Clinic Centre, Xi'an Chest Hospital, Xi 'an Jiaotong University Health Science Center, Xi’an, China

**Keywords:** MassARRAY, *mycobacterium tuberculosis*, drug resistance, gene mutation, diagnosis

## Abstract

**Introduction:**

*Mycobacterium tuberculosis* (MTB) identification and drug resistance diagnosis are very important for treatment of drug-resistant tuberculosis (DR-TB). Therefore, high throughput, accurate and low-cost molecular detection techniques are urgently needed. This study aimed to evaluate the clinical application value of MassARRAY in tuberculosis diagnosis and drug resistance screening.

**Methods:**

The limit of detection (LOD) and clinical application value of MassARRAY were evaluated using reference strains and clinical isolates. MTB in bronchoalveolar lavage fluid (BALF) and sputum samples were detected using MassARRAY, quantitative real-time polymerase chain reaction (qPCR) and MGIT960 liquid culture (culture). Using culture as the standard, the efficacy of MassARRAY and qPCR for the detection of TB was analyzed. Mutation of drug resistance genes in MTB clinical isolates was tested using MassARRAY, high-resolution melting curve (HRM), and Sanger sequencing. Using sequencing as the standard, the efficacy of MassARRAY, and HRM for the detection of each drug resistance site of MTB was analyzed. Simultaneously, the mutation of drug resistance genes by the MassARRAY method was compared with the results of drug susceptibility testing (DST), and the genotype–phenotype relationship was analyzed. The ability of MassARRAY to discriminate mixed infections was detected using mixtures of standard strains (M. tuberculosis H37Rv) and drug-resistant clinical isolates and mixtures of wild-type and mutant plasmids.

**Results:**

In MassARRAY, 20 related gene mutations could be detected by two PCR systems. All genes could be accurately detected when the bacterial load was 10^4^ CFU/mL. When the load of wild-type and drug-resistant MTB mixture was 10^5^ CFU/mL (respectively reached 10^4^ CFU/mL), variants and wild-type genes could be detected simultaneously. The sensitivity of MassARRAY (96.9%) for identification was higher than that of qPCR (87.5%) (*p* < 0.001). The sensitivity and specificity of MassARRAY for all drug resistance gene mutations were 100.0%, with higher accuracy and consistency than HRM (sensitivity = 89.3% and specificity = 96.9%, *p* = 0.001). Analyzing the relationship between MassARRAY genotype and DST phenotype, the accuracy of katG_315, rpoB_531, rpsL_43, rpsL_88, and rrs_513 sites was 100.0%, and embB_306 and rpoB_526 were inconsistent with the DST results when the base changes were different.

**Discussion:**

MassARRAY can obtain base mutation information and identify heteroresistance infections simultaneously when the mutant proportion was at least 5–25%. It has good application prospects in the diagnosis of DR-TB with high throughput, accurate and low-cost.

## 1. Introduction

Tuberculosis (TB) is a communicable disease, a major cause of ill health and one of the leading causes of death worldwide, with a mortality of 17/100, 000 (World Health Organization, 2022). In China, 16,826 people were diagnosed with drug-resistant TB (DR-TB) in 2021 (16,343 in 2020 ↑ + 3%; World Health Organization, 2022). TB is curable and preventable. About 85% of people who develop TB disease can be successfully treated with a 6-month drug regimen, and treatment for DR-TB takes at least 9–18 months (World Health Organization, 2022). These successful treatments depend largely on *Mycobacterium tuberculosis* (MTB) identification and drug resistance diagnosis.

Currently, the laboratory diagnosis of DR-TB mainly depends on traditional culture-dependent drug susceptibility testing (DST) and nucleic acid amplification testing (NAAT). The traditional DST covers more comprehensive drugs and can obtain accurate drug susceptibility information. However, it takes 1–2 months to obtain results due to the long growth cycle of MTB. Ineffective drug treatment for patients with DR-TB during this period will cause greater physical, psychological and economic pressure. Therefore, molecular diagnostic technology for rapid DR-TB diagnosis has been the main direction of research recently. Drug resistance is mediated by single-nucleotide polymorphisms (SNPs), multinucleotide polymorphisms, insertions and deletions (indels) and rearrangements in chromosomal genes that encode drug targets. The genotype–phenotype relationship associated with drug resistance varies depending on the type of genetic alteration. Nonsense mutations and genetic compensation response lead to a low correlation with bacterial resistance (Ma and Chen, 2019). Furthermore, heteroresistance is another factor complicating the diagnosis of DR-TB. It refers to the coexistence of susceptible and resistant MTB variants or multiple resistant strains with discrete resistance-conferring mutations within a single specimen (Koch et al., 2018). It leads to differences in detection performance among various types of NAAT in clinical practice. The Xpert system is a common molecular technology in clinical practice. It can obtain the results of MTB and rifampicin rpoB resistance within 2 h but cannot obtain the mutation type of gene loci. Bruker–Hain and gene chip technology are polymerase chain reaction-(PCR-) based probe hybridization techniques, which can obtain the results of gene site mutation of multiple anti-TB drugs. High-resolution melting (HRM) curve assay is a simple, rapid and cost-effective method that can be performed in a closed tube (Kohli et al., 2021). This method needs only the usual unlabeled primers and a double-stranded DNA-binding dye and detects sequence variants based on differences in the melting profiles between test and reference DNA. It can detect whether the target gene is mutated but cannot obtain specific information on the mutation site. Sanger sequencing technology is the gold standard for genetic testing but has low throughput. Next-generation sequencing (NGS) technology with high throughput characteristics is most certainly not a “user-friendly” technology, requires expensive instrumentation, bioinformatics and highly trained individuals, and therefore is not accessible to the majority of the clinical laboratories, particularly in countries with a high incidence of tuberculosis. Therefore, a high throughput, low-cost detection technique that can obtain accurate mutation sites is urgently needed to diagnose DR-TB.

The MassARRAY system is DNA time-of-flight mass spectroscopy developed by Sequenom, Inc. San Diego, United States. It can meet the above needs based on the advantages of its principle. Based on multiplex PCR, MassARRAY uses a single-base extension method similar to the Sanger method, uses dideoxynucleotides (ddNTPs) to extend a single base after the primer, and detects the mutation type through the difference in the mass of nucleotides (adenine, guanine, cytosine, and thymine). DNA time-of-flight mass spectroscopy is a flexible throughput ultrasensitive mutation detection system (Murray, 1996; Edwards et al., 2005; Tost and Gut, 2006; Yuan et al., 2011). Therefore, the detection of most TB drug resistance gene loci can be obtained within 1–2 reaction systems, and the accuracy is similar to that of the Sanger method in principle.

This study aimed to evaluate the application value of MassARRAY in the diagnosis of DR-TB by analyzing the limit of detection (LOD) and clinical application value.

## 2. Materials and methods

### 2.1. Subject and study design

Samples from patients with confirmed tuberculosis, including 52 bronchoalveolar lavage fluid (BALF), 42 sputum, and 128 MTB clinical isolates, were collected from Xi’an Chest Hospital. This study was approved by the Ethics Committee of Xi’an Chest Hospital [No: R2022-003-01]. Reference strains *Mycobacterium tuberculosis* H37Rv were obtained from the Chinese Center for Disease Control and Prevention, and CMCC95102/CMCC95103 were purchased from the National Center for Medical Culture Collections. Wild-type and mutant plasmid mixtures were synthesized by Sangon Biotech (Shanghai) Co., Ltd.

**Figure 1:**
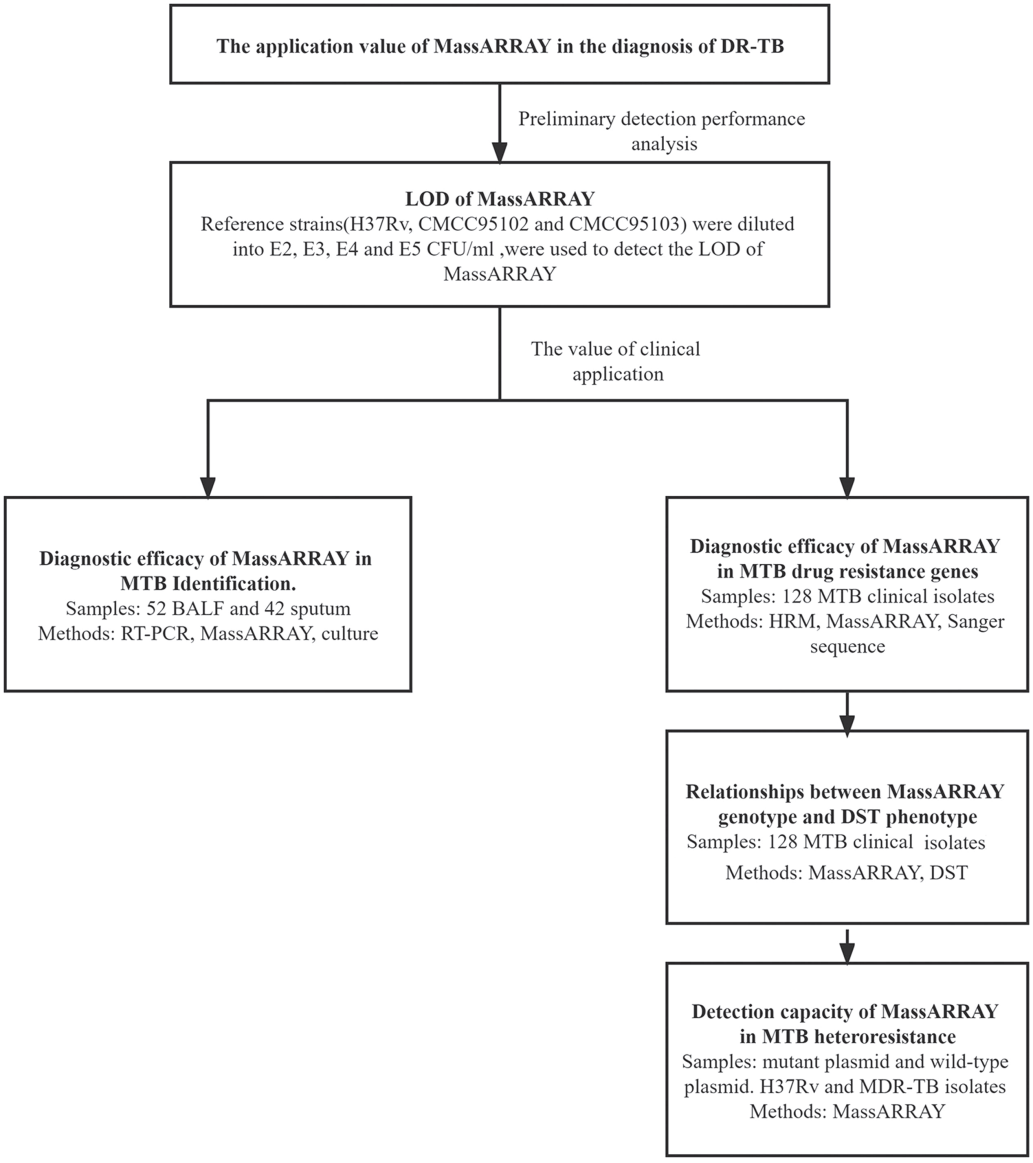
Design of experiment.

As illustrated in Figure 1, the application value of MassARRAY in the diagnosis of DR-TB was evaluated by preliminary detection performance analysis and the value of clinical application.

LOD of MassARRAY was conducted for preliminary detection performance analysis: the reference strains were diluted into 10^2^, 10^3^, 10^4^, and 10^5^ CFU/mL. Reference strain DNA was extracted using three commonly used clinical nucleic acid extraction methods (Magnetic bead method, Column extraction method, and Glass bead method), and was used to detect the LOD of MassARRAY for TB identification and drug resistance gene detection. The reference strains *M. tuberculosis* H37Rv, CMCC95102, and CMCC95103 were used to repeat the test. Two identification sites and 25 drug-resistant genes sites were detected by MassARRAY (Table 1). The lowest concentration that can be detected is LOD.

**Table 1:** Description of MassARRAY and HRM gene detection sites.

MassARRAY						HRM			
Reaction system, tube	Drug/identification	Resistance locus	Single base extension primer	Wild base	Mutant base	Reaction system,tube	Drug	Resistance locus	Wild melting temperature
M_1	SM	rrs_905 1,472,752	Forward-ACGTTGGATGGTGGGTTTCCTTCCTTGGGA	A	G	H_1	RFP	ropb507-512	66°C ± 1°C
M_1			Reverse-ACGTTGGATGCGTCCTGTGCATGTCAAACC			H_1		ropb521-528	74°C ± 1°C
M_1		rrs_513 1,472,359	Forward-ACGTTGGATGCGGATTGACGGTAGGTGGAG	A	C	H_2		ropb513-520	65°C ± 1°C
M_2			Reverse-ACGTTGGATGCGGATTGACGGTAGGTGGAG			H_2		ropb529-533	67°C ± 1°C
M_2		rpsL_88 781,822	Forward-ACGTTGGATGACCTGCAGGAGCACTCGAT	A	G/T	H_3	INH	AhpC(−44 ~ −30,-15 ~ 3)	58.1°C ± 1°C
M_2			Reverse-ACGTTGGATGACCGCGGATGATCTTGTAGC			H_3		inhA94	66°C ± 1°C
M_1		rpsL_43 781,687	Forward-ACGTTGGATGAAGGGTCGTCGGGACAAGAT	A	G/T	H_4		inhA(−17 ~ −8)	64°C ± 1°C
M_2			Reverse-ACGTTGGATGAAGGGTCGTCGGGACAAGAT			H_4		KatG315	68°C ± 1°C
M_2	RFP	rpoB_511 761,095	Reverse-ACGTTGGATGCGATCAAGGAGTTCTTCGGC	T	C	H_5	EMB	embB306	66.5°C ± 1°C
M_1		rpoB_516 761,109	Forward-ACGTTGGATGGGCACGCTCACGTGACAGA	G	T	H_5		embB497	61.5°C ± 1°C
M_1		rpoB_516 761,110	Reverse-ACGTTGGATGCGATCAAGGAGTTCTTCGGC	A	G/T	H_6		embB406	64.5°C ± 1°C
M_2		rpoB_526 761,139	Forward-ACGTTGGATGCGATCAAGGAGTTCTTCGGC	C	G/T/A	H_6		embB378	61.5°C ± 1°C
M_2		rpoB_526 761,140	Reverse-ACGTTGGATGGGCACGCTCACGTGACAGA	A	G/T	H_7	SM	rpsL43	57.0°C ± 1°C
M_1		rpoB_531 761,155	Forward-ACGTTGGATGCGATCAAGGAGTTCTTCGGC	C	T/G	H_7		rpsL88	65.0°C ± 1°C
M_1		rpoB_533 761,161	Forward-ACGTTGGATGGGCACGCTCACGTGACAGA	T	C	H_8		rrs513-517	63.5°C ± 1°C
M_1	INH	katG_315 2,155,168	Forward-ACGTTGGATGGGATCTCGAGGAAACTGTTG	G	C/A	H_8		rrs905-908	65.0°C ± 1°C
M_1		katG_315 2,155,169	Reverse-ACGTTGGATGTGGAAGAGCTCGTATGGCAC	C	A	H_9	FQ	gryA88 ~ 94	71.0°C ± 1°C
M_1		inhA-15C 1,673,425	Forward-ACGTTGGATGCTCGTGGACATACCGATTTC	C	T				
M_1			Reverse-ACGTTGGATGACTGAACGGGATACGAATGG						
M_2	EMB	embB_306 4,247,429	Forward-ACGTTGGATGATATTCGGCTTCCTGCTCTG	A	G/C				
M_2		embB_306 4,247,431	Reverse-ACGTTGGATGACCAGCGGAAATAGTTGGAC	G	A/C/T				
M_2	FQ	gyrA_94 7,581	Forward-ACGTTGGATGGAGCCGAAGTTGCCCTGG	G	A/C/T				
M_2		gyrA_94 7,582	Reverse-ACGTTGGATGATGCAATGTTCGATTCCGGC	A	G/C				
M_1		gyrB_499 6,620	Forward-ACGTTGGATGCGACCGCTTTTTGCAGAACC	G	A				
M_1			Reverse-ACGTTGGATGCGTAAGGCACGAGAGTTGGT						
M_1	Mycobacteria identification	IS6110	Reverse-ACGTTGGATGTACGTGGCCTTTGTCACCGA	C					
M_1		ext_rd9	Reverse-ACGTTGGATGTAGCCACCACCGACTCATAC	G					

Streptomycin, SM; Rifampicin, RFP; Isoniazid, INH; Ethambutol, EMB; Fluoroquinolone, FQ; HRM, △melting temperature ≥ 2°C is defined as mutation.

The value of clinical application: MTB in BALF and sputum samples were detected using MassARRAY, quantitative real-time polymerase chain reaction (qPCR) and MGIT960 liquid culture (culture). Using culture as the standard, the efficacy of MassARRAY and qPCR for the detection of TB was analyzed. Mutation of drug resistance genes in MTB clinical isolates was tested using MassARRAY, HRM, and Sanger sequencing. Using sequencing as the standard, the efficacy of MassARRAY, and HRM for the detection of each drug resistance site of MTB was analyzed. Simultaneously, the mutation of drug resistance genes by the MassARRAY method was compared with the results of DST, and the genotype–phenotype relationship was analyzed. The ability of MassARRAY to discriminate mixed infections was detected using mixtures of standard strains (*M. tuberculosis* H37Rv) and drug-resistant clinical isolates and mixtures of wild-type and mutant plasmids. The concentration of drug-resistant gene wild plasmid and mutant plasmid was 10^7^ copies/μL, mixed in different ratios, and the ratios of mutant plasmid was 5, 10, 15, 20, and 25%. *M. tuberculosis* H37Rv and clinical isolates of drug-resistant strains were diluted to 10^3^, 10^4^, 10^5^, and 10^6^ CFU/mL, mixed in different ratios, and the ratios of clinical isolates of drug-resistant strains was 20, 40, 60, and 80%.

### 2.2. DNA extraction

Magnetic bead method: Add 1 mL of bacterial culture solution to an EP tube, centrifuge at 12,000 × g for 5 min, and aspirate the supernatant as much as possible. Add 300 μL lysozyme solution (50 mg/mL) and resuspend. Heat at 99°C for 10 min. Then, transfer all the bacterial liquid to the first hole of the extraction kit. Use the Lab-aid 824 nucleic acid extraction system (Zeesan Biotech, Xiamen, China) to lyse, elute and extract DNA.

Column extraction method: Add 1 mL of bacterial culture solution to an EP tube, centrifuge at 12,000 × g for 5 min, and aspirate the supernatant as much as possible. Add 110 μL buffer (20 mmol /L Tris, pH 8.0; 2 mmol/L Na2-EDTA; 1.2% Triton), 70 μL lysozyme solution (50 mg/mL), and 4 μL Rnase A (100 mg/mL) solution at 37°C for more than 30 min. Shake for 15 s and leave at room temperature for 5 min. Add 20 μL Proteinase K solution to the tube and mix. Usethe TIANamp Bacteria DNA Kit (TIANGEN BIOTECH (BEIJING) CO., LTD) to lyse, elute and extract DNA.

Glass bead method: For Bacteria, add 1 mL bacterial liquid to a 1.5 mL EP tube with glass beads (acid-washed, 710–1,180 μm, G1152, 150–212 μm, G1145 (1: 3), Sigma), and centrifuge at 12,000 × g for 5 min, remove the supernatant; add and 50 μL nucleic acid extraction solution (10 mmol/L Tris–HCl Ph 8.0, 1% Triton X-100, 1 mmol/L EDTA-2Na) in the tube, then vortex and mix it for 10 min, heat at 95°C for 5 min, and centrifuge at 12,000 × g for 2 min. The supernatant was the template DNA. For BALF and sputum samples, an equal volume of 4% NaOH solution was added, shake and mix, leave at room temperature for 15 min. Pipette 1 mL of the mixture into a 1.5 mL EP tube, centrifuge at 12,000 × g for 5 min, discard the supernatant, add 1 mL of washing solution (5 mmol/L EDTA-2Na), shake and mix, centrifuge at 12,000 × g for 5 min, and discard the supernatant as much as possible. Add 50 μL nucleic acid extraction solution (10 mmol/L Tris–HCl pH 8.0, 1% Triton X-100, 1 mmol/L EDTA-2Na) to resuspend. Transfer the resuspension to a tube with glass beads (Sigma). The tube was vortexed and mixed for 10 min, heated at 95°C for 5 min, and centrifuged at 12,000 × g for 2 min. The supernatant can be used for PCR.

### 2.3. MassArray

MassArray was used to identify MTB and detect MTB resistance gene mutation sites. The anti-tuberculosis drugs and their corresponding gene resistance loci are shown in Table 1. Two PCR reaction systems were performed simultaneously. The reaction system contains HPLC H_2_O 0.8 μL, 10XPCR buffer with 20 mM MgCl_2_ 0.5 μL, 25 mM MgCl_2_ 0.4 μL, 25 mM dNTP Mix 0.1 μL, 0.5 μM Primer Mix 1 μL, PCR Enzyme 0.2 μL and 2 μL template DNA, in a final volume of 5 μ. The reaction program was: 95°C for 2 min; 95°C for 30s, 60°C for 30s, 72°C for 60s, for 45 cycles; 72°C for 5 min, hold at 4°C. Add 2 μL shrimp alkaline phosphatase (SAP) in each well, 37°C for 40 min, 85°C for 5 min, hold at 4°C. Add 2 μL iPLEX extension mix (nanopure water 0.62 μL, iPLEX buffer 0.2 μL, iPLEX termination mix 0.2 μL, extend primer mix 0.94 μL, and iPLEX enzyme 0.04 μL, Agena Bioscience, San Diego, CA) in each well. The reaction program was: 95°C for 30s; 95°C for 5 s, (52°C for 5 s, 80°C for 5 s, for 5 cycles), for 40 cycles; 72°C for 3 min, hold at 4°C.

The production was then carried out at Zhejiang Digena R&D Center, on a high-throughput MassARRAY platform with data analyzed using Typer 4.0 and plate manager 1.0 software. The quality of the test results was classified as No-Alleles, Low Probability, Aggressive, Moderate, Conservative.

### 2.4. Quantitative real-time PCR

DNA was extracted by Glass bead method (Method 2.2), 2 μL DNA was added into 18 μL amplification mixture (*Mycobacterium* nucleic acid detection kit (PCR - fluorescent probe method) (CapitalBio Technology, Beijing, China)), in a final volume of 20 μL. And PCR reaction was performed by ABI 7500 PCR instrument (Applied Biosystems Inc., United States). The reaction program was: 37°C, 300 s; 94°C, 180 s; 94°C, 15 s, 60°C, 30s for 40 cycles; 50°C, 10s. The fluorescence collection points were selected at 60°C, 30s. The target gene was IS6110.

### 2.5. Culture

BACTEC MGIT 960 liquid culture was used. Two milli liter samples were mixed with 4% NaOH solution at 1: 1 ~ 1: 2 and added into a 50 mL centrifuge tube. After shaking and fully liquifying, the samples were left standing for 15 min, and 0.1 mol/L phosphate buffer solution (PBS) was added to 45 mL. After centrifugation at 3,000 × g for 20 min, the supernatant was discarded, 1 mL 0.1 mol/L PBS was added and mixed, 0.5 mL was inoculated into the MGIT 960 culture tube (contains BBL MGIT nutritional supplement OADC and BBL MGIT miscellaneous bacteria inhibitor PANTA) (Becton, Dickinson and Company, United States) and placed in the BACTEC MGIT 960 incubator for 42 days culture. The instrument automatically reports the results.

### 2.6. High-resolution melting curve assay

High-resolution melting curve real-time PCR experiments were performed with the SLAN-96S Real-time fluorescence quantitative PCR detection system (Zeesan Biotech, Xiamen, China), using an MTB drug resistance mutation detection kit (Fluorescent PCR melting curve method, Zeesan Biotech, Xiamen, China). Nine PCR reaction systems were performed simultaneously, in a final volume of 25 μL containing 2 μL template DNA. The reaction program was: 50°C, 60s; 95°C, 600 s; 95°C, 15 s, 70°C, 20s (reduced 1°C for each cycle), 76°C, 25 s for 13 cycles; 95°C, 15 s, 57°C, 20s, 76°C, 25 s for 42 cycles; 95°C, 120 s; 40°C, 120 s; 45°C–85°C, fluorescence signals were collected every 1°C for this period. The anti-tuberculosis drugs and their corresponding gene resistance loci are shown in Table 1. The software analyzes the differences in the shape of the melting curve between a sample and the wild-type control strain (*M. tuberculosis* H37Rv) by generating a difference plot curve. This plot helps with clustering samples into groups having similar melting curves; hence, sequence polymorphisms can be detected.

### 2.7. Sanger sequence analysis

Primers of MassArray listed in Table 1 were used for Sanger sequencing. The test was performed by Sangon Biotech (Shanghai, China), sequence analysis was performed using BLAST,^1^ using *M. tuberculosis* H37Rv genome (NC_000962.3) as the reference sequence.

### 2.8. Drug susceptibility testing

Antibiotic susceptibility was tested using Middlebrook 7H11 agar media. The critical concentrations of each drug were listed below: Rifampicin (RFP) 1 μg/mL, Isoniazid (INH) 0.1 μg/mL, Ethambutol (EMB) 7.5 μg/mL, Streptomycin (SM) 2 μg/mL, Fluoroquinolone (FQ) 1 μg/mL. The positive culture was diluted to 10^3^ CFU/mL with Middlebrook 7H11 agar media and added to the drug plate. The results were reported after incubation at 37°C for 10–21 days.

### 2.9. Statistical analysis

SPSS 22.0 statistical software (IBM Inc. 2019, New York, United States) was used for data processing. Assuming the sensitivity of RT_PCR was 0.970 (Rozales et al., 2014), using a significance level of 5% (α) and 80% (δ) power, we calculated that 17 patients with culture positive tuberculous were required. To provide robust specificity (0.915) estimates, at least 47 patients with culture negative tuberculous was also tested. The sensitivity, specificity, PPV, NPV, accuracy, AUC, and Kappa value were calculated, *p* < 0.05 was considered statistically significant. The heat map and percent stacked column chart were performed by Graphpad Prism 7.04 (GraphPad Software, California, United States).

## 3. Results

### 3.1. Baseline characteristics of clinical participants

All biological samples in this study were collected from patients with TB diagnosed at Xi’an Chest Hospital from June 2021 to December 2021. The 94 clinical specimens contained 42 sputum and 52 BALF, and 128 MTB clinical isolates contained 30 multi-drug resistant (MDR) isolates, 21 Rifampicin-resistant (R-R) isolates, and 77 sensitive isolates. Table 2 shows the baseline characteristics of the participants.

**Table 2:** Baseline characteristics of tuberculosis patients in clinically isolated samples.

Baseline characteristics			Number
Clinical samples, *n* = 94			
	Sex		
		Male	48
		Female	46
	Age, IQR		39.50 (26.00,63.00)
	Sample type		
		Sputum	42
		BALF	52
Clinical bacteria, *n* = 128			
	sex		
		Male	83
		Female	45
	Age, IQR		47.50 (28.00,62.75)
	Drug resistance		
		MDR	30
		R-R	21
		Sensitive	77

### 3.2. LOD for MassARRAY

**Figure 2:**
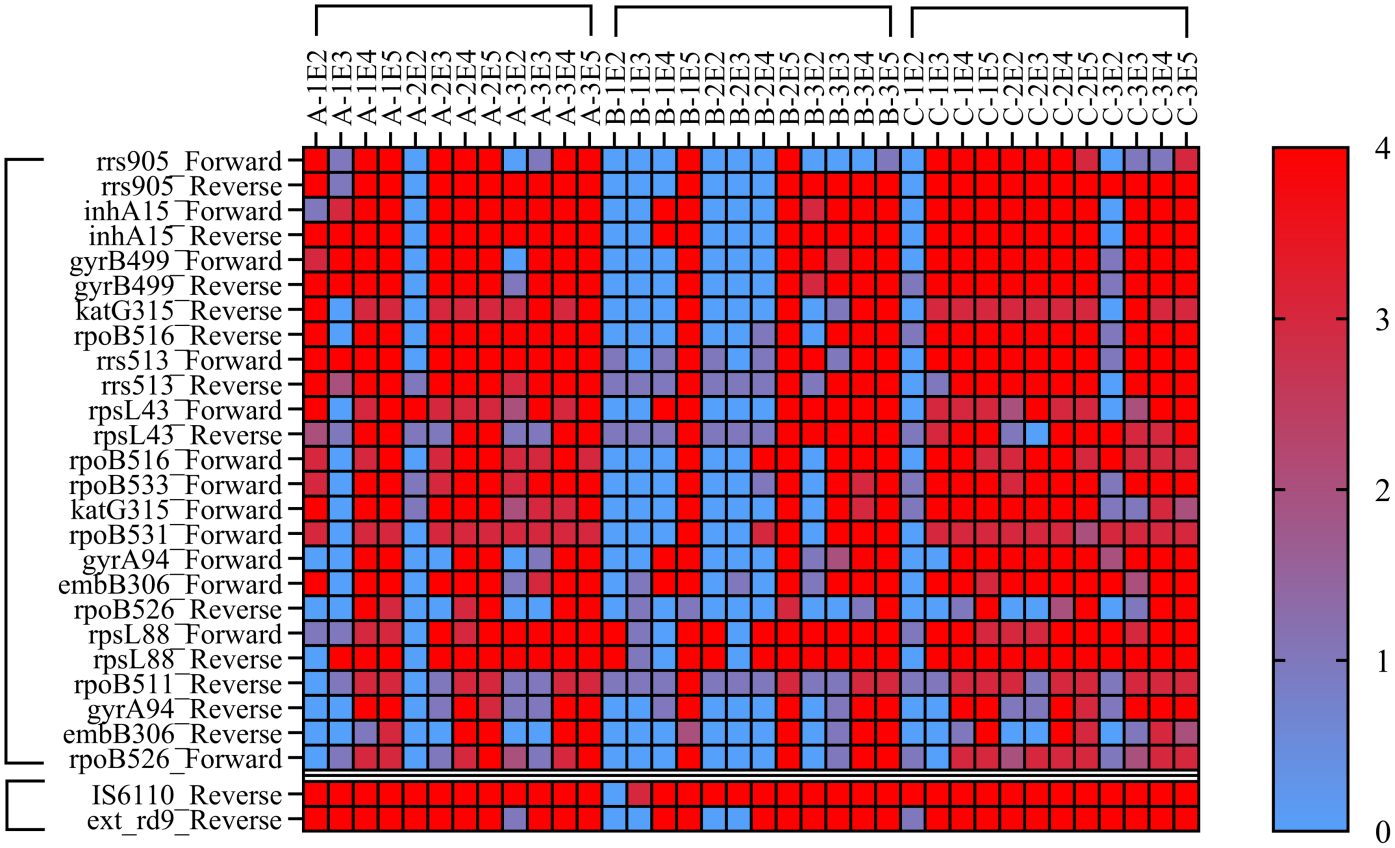
Heat Map of minimum detection limit for MassArray. Note: A = Magnetic bead method, B=Column extraction method, C = Glass bead method, 1, 2, 3 refer to H37Rv, CMCC95102, CMCC95103. E2-E5 refer to 10^2^–10^5^ CFU/mL. Each sample was tested by MassArray for three times, and the lowest detection value was selected for statistical analysis. 0–4 refer to the quality of the test results, 0 = No-Alleles, 1 = Low Probability, 2 = Aggressive, 3 = Moderate, 4 = Conservative. The value of 1 was used to determine the lowest bacterial load to trigger positive response.

DNA extracted by three nucleic acid extraction methods was used for MassARRAY LOD detection. The results (Figure 2) showed that LOD of Magnetic bead method and Glass bead method in two TB identification genes was 10^2^ CFU/mL, and Column extraction method was 10^4^ CFU/mL. In the 25 drug resistance gene sites detection, the LOD of Magnetic bead method and Glass bead method was 10^4^ CFU/mL, and Column extraction method was 10^5^ CFU/mL. When the bacterial concentration was lower than 10^4^ CFU/mL, the LOD of isoniazid, rifampicin resistance genes rpoB, KatG and inhA reached 10^2^ CFU/mL by glass bead method. The MassARRAY has a lower LOD using the Glass bead method for nucleic acid extraction.

### 3.3. Diagnostic efficacy of MassARRAY in MTB identification

As shown in Table 3, the positive rate of culture was 34.0% (32/94), MassARRAY was 51.1% (48/94), and qPCR was 40.4% (38/94). Of the 94 patients with confirmed TB, the positive rate of MassARRAY was 96.9% (31/32) in patients with culture-positive TB and 27.4% (17/62) in patients with culture-negative TB. Both were higher than those of qPCR (87.5% (28/32) and 16.1% (10/62)). Using culture as the standard, the overall sensitivity of MassARRAY was 96.9%, which was higher than that of qPCR (87.5%), and the sensitivity of each sample type was also higher than that of qPCR. Therefore, MassARRAY had higher diagnostic efficiency than the traditional qPCR technology.

**Table 3:** Detection efficiency of tuberculosis identification in clinical samples.

Method	Sample		Culture		*p* value	Sensitivity	Specificity	PPV	NPV	Accuracy	ROC	Kappa value
			*N*	*P*								
MassARRAY	BALF	N	25	1	–	94.1%	71.4%	61.5%	71.4%	78.8%	0.828	0.577
		P	10	16								
	Sputum	N	20	0	–	100.0%	74.1%	68.2%	74.1%	83.3%	0.870	0.671
		P	7	15								
	Total	N	45	1	–	96.9%	72.6%	64.6%	72.6%	80.9%	0.847	0.703
		P	17	31								
qPCR	BALF	N	31	1	<0.001	94.1%	88.6%	80.0%	88.6%	90.4%	0.913	0.791
		P	4	16								
	Sputum	N	21	3	<0.001	80.0%	77.8%	66.7%	77.8%	78.6%	0.789	0.553
		P	6	12								
	Total	N	52	4	<0.001	87.5%	83.9%	73.7%	83.9%	85.1%	0.857	0.683
		P	10	28								

N: Negative; P: Positive. qPCR detection site is Mycobacterium gene IS6110. The nucleic acid extraction method of MassARRAY and qPCR was Glass bead method. Chi-square test was used to compare the sensitivity of qPCR with that of MassARRAY.

### 3.4. Diagnostic efficacy of MassARRAY in MTB drug resistance genes

Using sequencing as the gold standard, MassARRAY has a sensitivity and specificity of 100.0% in the detection of all drug resistance gene mutations and has high accuracy and consistency, which is consistent with the results of sequencing. The sensitivity and specificity of drug-resistant mutations detected by HRM were 89.3 and 96.9%, respectively, which were lower than those of the MassARRAY method. The difference between HRM in the gyrA_94 gene and the other two methods was statistically significant (*p* = 0.006) because the results of sequencing and MassARRAY were consistent, and no statistically significant difference in other genes was observed (*p* > 0.05). Taking all 12 genes as a whole, the difference is still statistically significant (*p* = 0.001; Table 4).

**Table 4:** Analysis of the efficiency of MassARRAY for gene mutation detection.

Gene	Test		Sequencing		Total	Sensitivity	Specificity	PPV	NPV	Accuracy	AUC	Kappa value	*p* value
			W	M									
rpoB_511	Mass ARRAY	W	127	0	127	100.0%	100.0%	100.0%	100.0%	100.0%	1	1	1
		M	0	1	1								
	HRM	W	124	0	124	100.0%	97.6%	25.0%	97.6%	97.7%	0.988	0.392	0.25
		M	3	1	4								
rpoB_526	Mass ARRAY	W	118	0	118	100.0%	100.0%	100.0%	100.0%	100.0%	1	1	1
		M	0	10	10								
	HRM	W	116	3	119	70.0%	98.3%	77.8%	98.3%	96.1%	0.842	0.716	1
		M	2	7	9								
rpoB_516	Mass ARRAY	W	126	0	126	100.0%	100.0%	100.0%	100.0%	100.0%	1	1	1
		M	0	2	2								
	HRM	W	121	0	121	100.0%	96.0%	28.6%	96.0%	96.1%	0.980	0.431	0.063
		M	5	2	7								
rpoB_531	Mass ARRAY	W	106	0	106	100.0%	100.0%	100.0%	100.0%	100.0%	1	1	1
		M	0	22	22								
	HRM	W	105	1	106	95.5%	99.1%	95.5%	99.1%	98.4%	0.973	0.945	1
		M	1	21	22								
inhA_-15	Mass ARRAY	W	119	0	119	100.0%	100.0%	100.0%	100.0%	100.0%	1	1	1
		M	0	9	9								
	HRM	W	115	0	115	100.0%	96.6%	69.2%	96.6%	96.9%	0.983	0.802	0.125
		M	4	9	13								
KatG_315	Mass ARRAY	W	92	0	92	100.0%	100.0%	100.0%	100.0%	100.0%	1	1	1
		M	0	36	36								
	HRM	W	90	6	96	83.3%	97.8%	93.8%	97.8%	93.8%	0.906	0.840	0.289
		M	2	30	32								
embB_306	Mass ARRAY	W	116	0	116	100.0%	100.0%	100.0%	100.0%	100.0%	1	1	1
		M	0	12	12								
	HRM	W	115	0	115	100.0%	99.1%	92.3%	99.1%	99.2%	0.996	0.956	1
		M	1	12	13								
rpsL_43	Mass ARRAY	W	88	0	88	100.0%	100.0%	100.0%	100.0%	100.0%	1	1	1
		M	0	40	40								
	HRM	W	85	3	88	92.5%	96.6%	92.5%	96.6%	95.3%	0.945	0.891	1
		M	3	37	40								
rpsL_88	Mass ARRAY	W	121	0	121	100.0%	100.0%	100.0%	100.0%	100.0%	1	1	1
		M	0	7	7								
	HRM	W	120	2	122	71.4%	99.2%	83.3%	99.2%	97.7%	0.853	0.757	1
		M	1	5	6								
rrs_513	Mass ARRAY	W	126	0	126	100.0%	100.0%	100.0%	100.0%	100.0%	1	1	1
		M	0	2	2								
	HRM	W	124	0	124	100.0%	98.4%	50.0%	98.4%	98.4%	–	0.660	0.5
		M	2	2	4								
rrs_905	Mass ARRAY	W	128	0	128	-	100.0%	-	100.0%	100.0%	1	1	–
		M	0	0	0								
	HRM	W	120	0	120	-	93.8%	0	93.8%	93.8%	–	0	–
		M	8	0	8								
gyrA_94	Mass ARRAY	W	120	0	120	100.0%	100.0%	100.0%	100.0%	100.0%	1	1	1
		M	0	8	8								
	HRM	W	109	1	110	87.5%	90.8%	38.9%	90.8%	90.6%	0.892	0.495	0.006
		M	11	7	18								
total	Mass ARRAY	W	1,387	0	1,387	100.0%	100.0%	100.0%	100.0%	100.0%	1	1	1
		M	0	149	149								
	HRM	W	1,344	16	1,360	89.3%	96.9%	75.6%	96.9%	96.2%	0.931	0.797	0.001
		M	43	133	176								

W: Wild; M: Mutant.

### 3.5. Relationship between MassARRAY genotype and DST phenotype

The gene mutations of some drugs were highly correlated with the phenotype, such as katG_315, rpoB_531, rpsL_43, rpsL_88, and rrs_513, as shown in Table 5. In embB, gyrA_94 and rpoB_526, different single-base mutation types correlate differently with the phenotype. For example, embB_306ATG > GTG(G) mutation was consistent with phenotypic results (Accuracy: 100.0%); the correlation is poor in ATG > ATA(A) and ATG > ATC(C) (Accuracy: 25.0 and 50.0%); the phenomenon of poor correlation also appears in rpoB_526 CAC > AAC(A) (Accuracy: 33.3%); rpoB_526CAC > CTC(T), GAC(G), TAC(T) mutation was consistent with phenotypic results (Accuracy: 100.0%).

**Table 5:** Relationship between MassARRAY genotype and DST phenotype.

Drugs	Gene mutation type	DST			Accuracy
		*R*	*S*	Total	
EMB	Wild	8	108	116	93.1%
	embB_306ATG > ATA(A)	1	3	4	25.0%
	embB_306ATG > ATC(C)	1	1	2	50.0%
	embB_306ATG > GTG(G)	6	0	6	100.0%
	Total	16	112	128	90.6%
FQ	Wild	6	114	120	95.0%
	gyrA_94GAC > GGC(G)	5	2	7	71.4%
	gyrA_94GAC > TAC(T)	1	0	1	100.0%
	Total	12	116	128	93.8%
INH	Wild	6	78	82	95.1%
	inhA-15C > T(T)	6	2	9	66.7%
	katG_315AGC > ACC(C)	35	0	36	97.2%
	inhA-15C > T(T)&katG_315AGC > ACC(C)	1	0	1	100.0%
	Total	48	80	128	93.8%
RIF	Wild	3	90	93	96.8%
	rpoB_511CTG > CCG(T)	0	1	1	0
	rpoB_516GAC > GTC(T)	2	0	2	100.0%
	rpoB_526CAC > AAC(A)	1	2	3	33.3%
	rpoB_526CAC > CTC(T)	2	0	2	100.0%
	rpoB_526CAC > GAC(G)	3	0	3	100.0%
	rpoB_526CAC > TAC(T)	2	0	2	100.0%
	rpoB_531TCG > TTG(T)	21	0	21	100.0%
	rpoB_533CTG > CCG(C)	1	0	1	100.0%
	Total	35	93	128	95.3%
SM	Wild	2	77	79	97.5%
	rpsL_43AAG > AGG(G)	40	0	40	100.0%
	rpsL_88AAG > AGG(G)	7	0	7	100.0%
	rrs_513A > C(C)	2	0	2	100.0%
	Total	51	77	128	98.4%

### 3.6. Detection capability of MassARRAY in MTB heteroresistance

**Figure 3:**
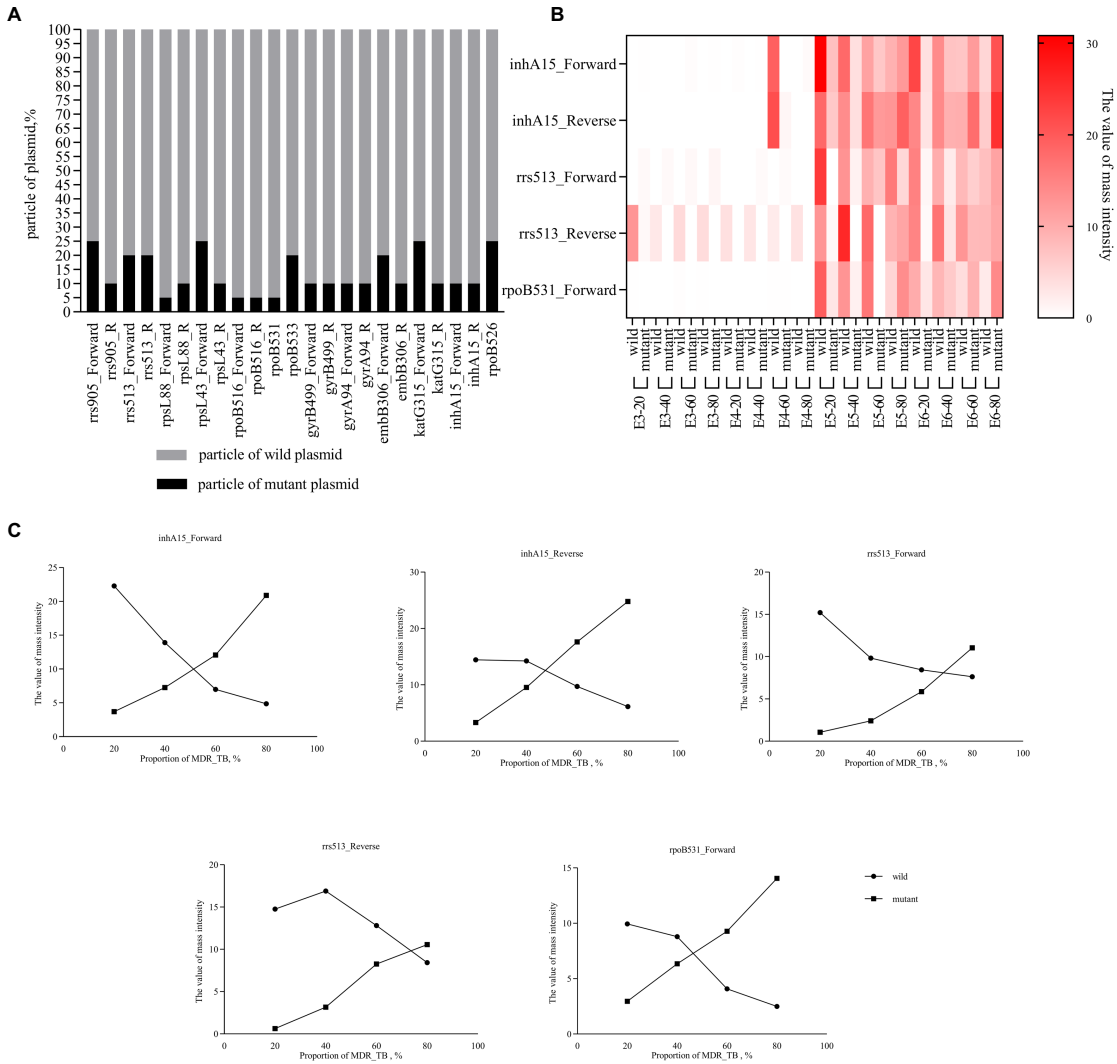
Detection capability of heteroresistance. **(A)** Percent stacked Chart of detection capability of heteroresistance by plasmid. The plasmid concentration is 10^7^ copies/μL, mixed in different ratios, and the final volume is 100 μL. **(B)** Heat map of detection capability of heteroresistance by bacteria. Clinically isolated MDR-TB was mixed with H37Rv at a ratio of 20–80%, the concentration was E3-E6 (10^3^–10^6^ CFU/mL), and the final volume was 1 mL. For example, E3-20 refers to concentration of mixture was 10^3^ CFU/mL (20% of Clinically isolated MDR-TB and 80% of H37Rv. **(C)** Line chart of mass intensity values in different proportions of MDR_TB. The concentration of the bacterial mixture in the Line chart of mass intensity was 10^5^ CFU/mL.

The resistance gene mutant plasmid and wild-type plasmid were mixed in different proportions. MassARRAY results showed that mutant and wild-type plasmids could be detected simultaneously when the mutant proportion was at least 5–25% (Figure 3A). H37Rv and MDR-TB isolates were mixed in different proportion,. MassARRAY results showed that variants and wild-type genes could be detected simultaneously when the mixture concentration was 10^5^ CFU/mL (respectively reached 10^4^ CFU/mL). The detection efficiency of some drug resistance genes decreased when the concentration was lower than 10^5^ CFU/mL (Figure 3B). Additionally, the value of mass intensity for variants was positively correlated with the concentration of MDR-TB, and the signal of mass intensity was not disturbed by the ratio of MDR-TB to H37Rv (Figure 3C).

## 4. Discussion

The “End TB Strategy” was developed by the World Health Organization (WHO) to end the global TB epidemic by 2035 (Uplekar et al., 2015). Therefore, the main development direction of early diagnostic technology for DR-TB is molecular diagnostic technology. DNA time-of-flight mass spectroscopy is a multiplexed ultrasensitive mutation detection system with a flexible throughput (Tost and Gut, 2006; Yuan et al., 2011).

Our study shows that MassARRAY has a low detection limit for MTB detection. Among the detection methods recommended by the WHO, the Gene Xpert MTB/RIF and Xpert MTB/RIF Ultra systems have the lowest LOD for the detection of MTB, which is significantly higher than that of other detection methods and can reach 10^2^ CFU/mL, and the rpoB gene can be detected at more than 200 CFU/mL (Balcha et al., 2014; Chakravorty et al., 2017; Tadesse et al., 2019; Kohli et al., 2021). In this study, the LOD of IS6110 and the rpoB gene by MassARRAY was also lower than 10^2^ CFU/mL, which indicated that it had a lower LOD and was superior to the existing molecular biology methods. Su et al.’s results show that the MALDI-TOF MS detection limit is less than 10 MTB copies (Su et al., 2017). The main reasons for the different LOD of NAAT are the nucleic acid extraction efficiency and amplification efficiency. This study compared three common clinical nucleic acid extraction methods. The glass bead method has the highest nucleic acid extraction efficiency. Previous studies reported that the method of physically breaking the wall could release bacterial nucleic acid more effectively because MTB has a thicker cell wall, thereby improving the detection rate (Afghani and Stutman, 1996; El-Hajj et al., 2001; Griffiths et al., 2006). In terms of amplification efficiency, MassARRAY uses multiplex PCR amplification for 45 cycles and single-base extension for 40 cycles, which makes the amplification efficiency of template DNA significantly higher than that of conventional PCR technology (mostly within 40 cycles). In addition, our results show that MassARRAY is more sensitive than qPCR (Table 3). Especially in culture-negative specimens, the positive rate of MassARRAY was significantly higher than that of qPCR, and more information of MTB resistance genes could be obtained.

The ability to accurately identify mutation types can obtain reliable molecular drug susceptibility results and provide a reference for clinical antibiotic treatment. The factors affecting the identification ability of experimental methods mainly come from two aspects: one is the defect of the method, and the other is derived from the microorganism. Mutation detection methods can be roughly divided into indirect and direct detection based on methodological principles. Indirect detection methods include Xpert MTB/RIF and HRM assay. Xpert uses five differently colored molecular beacons. It does not bind to its target if the target sequence differs from the rifampin-susceptible sequence by as little as a single-nucleotide substitution (El-Hajj et al., 2001). HRM is based on differences in the melting profiles of test and reference DNA (Keikha and Karbalaei, 2021). These two methods can quickly obtain the mutation information of drug resistance genes but cannot obtain the exact mutation type and cannot distinguish the gene-phenotype relationship. Direct detection methods include molecular probe-based hybridization technology and sequencing methods. Their advantage is that they can obtain accurate mutation types, and the results are more reliable than those of the indirect detection methods (Gupta and Kakkar, 2018). In this study, MassARRAY has the same accuracy as the gold standard Sanger sequencing. This finding is because MassARRAY and Sanger sequencing are based on single-base extensions after PCR, where Sanger sequencing detects the fluorescent signal marker ddNTP and MassARRAY detects molecular mass. Therefore, nucleic acid mass spectrometry is often referred to as mass spectrometry-based sequencing. Although HRM was not statistically different from sequencing in the detection of a single mutation site, this difference was statistically significant in the overall results for all gene mutation sites, as shown in Table 4. Genetic mutations are one of the causes of drug resistance in bacteria. There are many gene loci affecting phenotype, and gene mutation may not cause phenotypic resistance, which may be related to nonsense mutation and genetic compensation response. This study (Table 5) and related studies showed that the genotype–phenotype relationship is complex (Dookie et al., 2018). For example, rpoB_526CAC > CTC(T), GAC(G) and TAC(T) were more correlated with the phenotype, while rpoB_526CAC > AAC(A) showed poor correlation. Clinicians can choose the appropriate treatment based on this level of correlation, and high-correlation mutational outcomes can provide stronger evidence when choosing treatment with resistant regimens. However, a bacterial infection in the body may be heterogeneous, and the coexistence of sensitive and drug-resistant strains is a challenge to molecular drug resistance diagnosis. The focus of Xpert MTB/RIF and hybridization technology (such as Bruker–Hain) is whether the wild-type gene is detected. When the wild-type gene is not detected, it is interpreted as a mutation, which makes it impossible to identify drug-resistant bacteria in mixed infections. Mixed infections can be distinguished when two different melting temperatures are detected by the HRM method (Keikha and Karbalaei, 2021). MassARRAY has the same ability. During mass spectrometry detection, different single-base extension products will generate different mass intensities due to the mass difference of ddNTPs (adenine, guanine, cytosine and thymine). The value of mass intensity can reflect the bacterial load of wild-type and drug-resistant strains to distinguish mixed infection. This was verified in Figure 3C. The mixing of wild-type and mutant resistance genes did not cause interference between peak types, and the detection results will not be affected, only limited by the LOD. Therefore, MassARRAY can not only accurately obtain the mutation results and mutation types, but also differentiate heterogeneity of drug resistance genes.

The WHO has classified TB drug resistance gene diagnosis into low complexity automated NAAT (fully automatic, such as Xpert), moderate complexity automated NAAT (dye-labeled probe-based PCR, such as HRM) and high complexity reverse hybridization-based NAAT (complex hybridization-based technology, such as line probe assay) (World Health Organization, 2021). Based on this classification, MassARRAY can be considered a moderate complexity automated NAAT based on PCR. MassARRAY and HRM use nucleic acid mass and dye-labeled probes to detect variants, respectively. Compared with dye-labeled probe assay, the mass spectroscopy technique has certain advantages in practice. Dye-labeled probes are limited by the number of fluorescent dyes and fluorescent channels of the PCR instrument. Table 1 shows that HRM requires nine reaction systems to detect 17 mutation sites of five drugs, while MassARRAY requires only two reaction systems to detect more than 20 sites. The reaction systems of dye-labeled probe assay will increase with the increase of detection sites, while a similar phenomenon rarely occurs with MassARRAY. After amplification, different target gene segments have obvious mass differences due to different contents of adenine, guanine, cytosine and thymine, so 1–2 reaction systems can meet more detection sites. NAAT detection flux is mainly limited to instruments and reaction systems. For example, in this study, HRM and MassARRAY required nine and two reaction systems for one sample, respectively. This means that MassARRAY can complete the PCR amplification of more samples at the same PCR instrument. MassARRAY can complete the detection of 2 identified genes and 25 drug-resistant gene loci within 24 h, saving at least 9/2 times of reagent cost compared with HRM. The MassARRAY system has 384 wells, and the detection flux of time-of-flight mass spectrometry is more flexible and theoretically can complete the detection of 1–384 samples and save time and reagent cost.

MassARRAY is an advanced and relatively complex detection method. Its complexity mainly comes from the design of primers and PCR reaction systems. A reasonable primer design can increase detection accuracy and reduce the reaction system. Additionally, MassARRAY can only detect known mutation sites and cannot predict unknown types of mutations. However, for clinical detection, we need obvious mutation sites with high drug correlation to guide clinicians’ treatment, so this limitation does not affect its application in clinical practice. With the same accuracy as sequencing, it can save the cost of expensive sequencing chips compared with high throughput NGS and does not increase the financial burden on patients. The drug resistance sites targeted in this study are traditional anti-TB drugs, but the use of this method is not limited to these genes, and the resistance genes of anti-TB new drugs can be added to the system of our current study. Due to the short time of clinical application of the new drugs (Badaquiline and Delamanid) and the lack of clinical data, new drugs have not been involved in this study.

In conclusion, the MassARRAY system is a technology that can obtain accurate mutation information of a large number of drug resistance genes and identify heteroresistance infections. Simultaneously, it has the characteristics of low LOD, simple operation and process, high throughput and low detection cost. It has good application prospects in the diagnosis of clinical DR-TB, especially suitable for specialized TB hospitals.

## Data availability statement

The original contributions presented in the study are included in the article/supplementary material, further inquiries can be directed to the corresponding authors.

## Ethics statement

The studies involving human participants were reviewed and approved by the Ethical Committee of Xi’an Chest Hospital, Xi’an Chest Hospital. The patients/participants provided their written informed consent to participate in this study.

## Author contributions

HY, LD, and TZ designed the study and the questionnaire. HY, AL, JL, and TK designed the experiments and analyzed the results. YZ, YY, and FR provided clinical samples and ethical reviews. HY and JM performed statistical analyses. HY and AL wrote the manuscript. TZ contributed critically revised the manuscript. All authors contributed to the article and approved the submitted version.

## Funding

This work was supported by the Xi’an Innovation Ability Foundation Project-Medical Research Project [grant number: 21YXYJ0001]; the Xi’an Innovation Ability Foundation Project-Medical Research Project [grant number: 2021JH-04-0179]; Xi’an Health Commission - General research project [grant number: 2021yb13] and Key Research and Development Program of Shaanxi Province [grant number: 2020SF-109].

## Conflict of interest

TK was employed by Department of Reagent, Zhejiang Digena Diagnosis Technology CO., LTD, Zhejiang, China.

The remaining authors declare that the research was conducted in the absence of any commercial or financial relationships that could be construed as a potential conflict of interest.

## Publisher’s note

All claims expressed in this article are solely those of the authors and do not necessarily represent those of their affiliated organizations, or those of the publisher, the editors and the reviewers. Any product that may be evaluated in this article, or claim that may be made by its manufacturer, is not guaranteed or endorsed by the publisher.

## Footnotes

^1^ https://blast.ncbi.nlm.nih.gov

## References

[ref1] Afghani B. Stutman H. R. (1996). Polymerase chain reaction for diagnosis of *M. tuberculosis*: comparison of simple boiling and a conventional method for DNA extraction. Biochem. Mol. Med. 57, 14–18. doi: 10.1006/bmme.1996.0003, PMID: 8812720

[ref2] Balcha T. T. Sturegard E. Winqvist N. Skogmar S. Reepalu A. Jemal Z. H.. (2014). Intensified tuberculosis case-finding in HIV-positive adults managed at Ethiopian health centers: diagnostic yield of Xpert MTB/RIF compared with smear microscopy and liquid culture. PLoS One 9:e85478. doi: 10.1371/journal.pone.0085478, PMID: 24465572 PMC3899028

[ref3] Chakravorty S. Simmons A. M. Rowneki M. Parmar H. Cao Y. Ryan J.. (2017). The new Xpert MTB/RIF ultra: improving detection of mycobacterium tuberculosis and resistance to rifampin in an assay suitable for point-of-care testing. MBio 8:e00812–17. doi: 10.1128/mBio.00812-17 28851844 PMC5574709

[ref4] Dookie N. Rambaran S. Padayatchi N. Mahomed S. Naidoo K. (2018). Evolution of drug resistance in mycobacterium tuberculosis: a review on the molecular determinants of resistance and implications for personalized care. J. Antimicrob. Chemother. 73, 1138–1151. doi: 10.1093/jac/dkx506, PMID: 29360989 PMC5909630

[ref5] Edwards J. R. Ruparel H. Ju J. (2005). Mass-spectrometry DNA sequencing. Mutat. Res. 573, 3–12. doi: 10.1016/j.mrfmmm.2004.07.021 15829234

[ref6] El-Hajj H. H. Marras S. A. Tyagi S. Kramer F. R. Alland D. (2001). Detection of rifampin resistance in mycobacterium tuberculosis in a single tube with molecular beacons. J. Clin. Microbiol. 39, 4131–4137. doi: 10.1128/JCM.39.11.4131-4137.2001, PMID: 11682541 PMC88498

[ref7] Griffiths L. J. Anyim M. Doffman S. R. Wilks M. Millar M. R. Agrawal S. G. (2006). Comparison of DNA extraction methods for Aspergillus fumigatus using real-time PCR. J. Med. Microbiol. 55, 1187–1191. doi: 10.1099/jmm.0.46510-0, PMID: 16914647

[ref8] Gupta S. Kakkar V. (2018). Recent technological advancements in tuberculosis diagnostics - a review. Biosens. Bioelectron. 115, 14–29. doi: 10.1016/j.bios.2018.05.017, PMID: 29783081

[ref9] Keikha M. Karbalaei M. (2021). High resolution melting assay as a reliable method for diagnosing drug-resistant TB cases: a systematic review and meta-analysis. BMC Infect. Dis. 21:989. doi: 10.1186/s12879-021-06708-1, PMID: 34551717 PMC8456628

[ref10] Koch A. Cox H. Mizrahi V. (2018). Drug-resistant tuberculosis: challenges and opportunities for diagnosis and treatment. Curr. Opin. Pharmacol. 42, 7–15. doi: 10.1016/j.coph.2018.05.013, PMID: 29885623 PMC6219890

[ref11] Kohli M. Schiller I. Dendukuri N. Yao M. Dheda K. Denkinger C. M.. (2021). Xpert MTB/RIF ultra and Xpert MTB/RIF assays for extrapulmonary tuberculosis and rifampicin resistance in adults. Cochrane Database Syst. Rev. 2021:CD012768. doi: 10.1002/14651858.CD012768.pub3, PMID: 33448348 PMC8078545

[ref12] Ma Z. P. Chen J. (2019). Nonsense mutations and genetic compensation response. Yi Chuan 41, 359–364. doi: 10.16288/j.yczz.19-101, PMID: 31106771

[ref13] Murray K. K. (1996). DNA sequencing by mass spectrometry. J. Mass Spectrom. 31, 1203–1215. doi: 10.1002/(SICI)1096-9888(199611)31:11<1203::AID-JMS445>3.0.CO;2-3 8946729

[ref14] Rozales F. P. Machado A. B. De Paris F. Zavascki A. P. Barth A. L. (2014). PCR to detect mycobacterium tuberculosis in respiratory tract samples: evaluation of clinical data. Epidemiol. Infect. 142, 1517–1523. doi: 10.1017/S0950268813002598, PMID: 24107314 PMC9151241

[ref15] Su K. Y. Yan B. S. Chiu H. C. Yu C. J. Chang S. Y. Jou R.. (2017). Rapid sputum multiplex detection of the *M. tuberculosis* complex (MTBC) and resistance mutations for eight antibiotics by nucleotide MALDI-TOF MS. Sci. Rep. 7:41486. doi: 10.1038/srep41486, PMID: 28134321 PMC5278408

[ref16] Tadesse M. Abebe G. Bekele A. Bezabih M. Yilma D. Apers L.. (2019). Xpert MTB/RIF assay for the diagnosis of extrapulmonary tuberculosis: a diagnostic evaluation study. Clin. Microbiol. Infect. 25, 1000–1005. doi: 10.1016/j.cmi.2018.12.018 30583052

[ref17] Tost J. Gut I. G. (2006). DNA analysis by mass spectrometry-past, present and future. J. Mass Spectrom. 41, 981–995. doi: 10.1002/jms.1096, PMID: 16921576

[ref18] Uplekar M. Weil D. Lonnroth K. Jaramillo E. Lienhardt C. Dias H. M.. (2015). WHO's new end TB strategy. Lancet 385, 1799–1801. doi: 10.1016/S0140-6736(15)60570-0 25814376

[ref19] World Health Organization. WHO consolidated guidelines on tuberculosis: Module 3: Diagnosis - rapid diagnostics for tuberculosis detection. Geneva (2021). Available at: https://www.who.int/publications/i/item/9789240029415 (Accessed July 7, 2021). 34314130

[ref20] World Health Organization. Global tuberculosis report (2022). Available at: https://www.who.int/teams/global-tuberculosis-programme/tb-reports/global-tuberculosis-report-2022 (Accessed October 27, 2022).

[ref21] Yuan G. Zhang Q. Zhou J. Li H. (2011). Mass spectrometry of G-quadruplex DNA: formation, recognition, property, conversion, and conformation. Mass Spectrom. Rev. 30, 1121–1142. doi: 10.1002/mas.20315, PMID: 21520218

